# Alcohol Use and Violence Among Young Adults

**Published:** 2004

**Authors:** Brian M. Quigley, Kenneth E. Leonard

**Affiliations:** Brian M. Quigley, Ph.D., is a principal investigator at the Research Institute on Addictions, University at Buffalo, State University of New York, Buffalo, New York. Kenneth E. Leonard, Ph.D., is research professor of psychiatry, University at Buffalo, School of Medicine, and senior research scientist at the Research Institute on Addictions, University at Buffalo, State University of New York, Buffalo, New York

Approximately 40 percent of people experiencing violence are young adults ages 18 to 30; this translates into a greater risk for violence in this age group than in any other segment of the population (Perkins 1997). (Only 50 percent of these violent crimes are reported to the police, however [see Hart and Rennison 2003].) Leonard and colleagues (2002) found that 44 percent of men ages 18 to 30 in a community sample reported having experienced physical aggression, either as the target or initiator of aggression, in the past year. Among women of this age in the same sample, 28 percent reported experiencing some form of physical aggression in the past year. In a separate sample of college students (Leonard et al. 2002), 33 percent of males and 22 percent of females reported experiencing physical aggression in the past year.

Two locations—bars and homes—stood out as the most likely settings for violence (Leonard et al. 2002). Men were more likely to be the target of severe violence in bars: 30 percent of the most severe incidents involving men as victims, from both the general population and college samples, occurred in or around a bar. For women, bars were less frequently the scene of severe violent victimization: 22 percent of the most severe episodes reported by women in the general population sample and 23 percent of the most severe episodes reported by women in the college sample occurred in or around a bar. Women were more likely to be targets of severe violence at home (50 percent of the most severe episodes reported by women in the community sample and 63 percent of the most severe episodes reported by women in the college sample occurred in the home). Fewer men reported experiencing their most severe episode of violence at home (16 percent of the most severe episodes reported by men in the community sample and 31 percent of those reported by men in the college sample occurred in the home).

Laboratory research demonstrates that intoxicated people are more aggressive than sober people (Bushman 1997). Thus, the link between drinking in bars or at home and incidents of aggression is an important area of research. This sidebar examines the role that alcohol may play in violence at these locations.

## Violence in Bars

The Bar Violence Study, conducted in Buffalo, New York, was designed to systematically examine the putative causes of bar violence, both direct and indirect. These causes included individual differences among people who frequent bars, characteristics of bars at which violence occurs, and the situations preceding the violent incidents. Participants, who were between the ages of 18 and 30, included 194 men and 106 women who reported experiencing bar violence, 121 men and 106 women who had observed but did not experience violence, and 54 men and 60 women who frequented bars but had neither seen nor experienced violence in a bar. After administering a battery of individual difference and alcohol use tests to the participants, researchers interviewed them about the characteristics of their usual bars. If a subject reported experiencing violence, he or she was asked about the characteristics of the bar in which the violence had occurred and about the violent incident itself.

An examination of the violent incidents suggested that although drinking played a role, it did not appear to be a direct cause of the violence (Leonard et al. 2003*a*). Participants who initiated or were the victims of a violent event had not consumed more alcohol at the time of the event than had participants reporting only threatening events that did not result in violence. Alcohol consumption was related to the risk of injury, however. Among men who became involved in a violent bar event, the more drinks they had consumed, the more severe the injury to themselves as well as to the other person involved; the more highly intoxicated the other person involved in the violence was reported to be, the less severe the injury that the men reported experiencing themselves (Leonard et al. 2003*a*). This finding supports the hypothesis that alcohol is a facilitator rather than an instigator of aggressive behavior. It also is consistent with the view that the psychopharmacological effects of intoxication on decisionmaking may make a bad situation worse.

Not everyone who drinks at a bar experiences violence, and those who do become involved in violence often have unique personality characteristics and alcohol usage patterns. For example, men who had committed or been the target of a violent act in a bar scored higher on measures of anger-proneness and impulsiveness than did men who had not experienced bar violence (although they might have observed it). Men who had experienced violence also scored lower on measures of personality agreeableness. Men who had been involved in bar violence reported drinking more than men who had not been involved (i.e., nonviolent men); they also reported having more alcohol problems than nonviolent men.

A key difference between women’s and men’s experience in bars lies in the types of aggression they encounter. Parks (2000) found that approximately one-third of the aggression experienced by women in bars was sexual in nature, involving behaviors ranging from inappropriate comments to nonconsensual physical contact.

Women who experienced violence in bars were found to consume more alcohol in general and score higher in anger-proneness than women who did not (Leonard et al. 2003*b*). This study also found that women who experienced severe violence in a bar had consumed more alcohol at the bar and were more likely to go to the bar alone or leave alone or with a stranger.

Bars’ social and environmental characteristics also were examined in the Bar Violence Study (Quigley et al. 2003). The clientele of violent bars tended to be younger and more likely to score higher on measures of anger and impulsiveness. They also were heavier drinkers, more likely to have alcohol problems, and more likely to believe that alcohol increases aggression. In addition, participants in the study were more likely to report that violent bars were smokier, higher in temperature, dirtier, darker, more crowded, more likely to have competitive games, and more likely to employ bouncers and male employees. As Buddie and Parks found in their research on women’s violent experiences in bars (2003), bars where violence occurred tended to be more permissive of clients’ displays of antinormative behavior, including sexual behavior and illegal activities. Based on regression analyses in which the effects of clientele factors were not significant predictors of bar status (violent or not) when bar characteristics were entered into the equation, the characteristics of the bars rather than the characteristics of the clientele seem to be the stronger determinant of whether violence occurred or not. However, people with certain characteristics may be attracted to certain types of bars, or the characteristics of the bars may be driven by their type of clientele—the exact relationship remains unclear.

Nevertheless, addressing the distinctive social and physical characteristics of violent bars may help reduce bar violence. The Safer Bars Program (Graham et al. 2004) attempted to decrease violence by training bar staff in numerous areas that could impact the potential for violence, such as serving practices and ways to defuse conflict situations, and by helping them to recognize physical and situational characteristics of bars that might promote or result in violence. Following training of employees at 18 violent bars, independent observations demonstrated that these bars had fewer instances of severe aggression by patrons and fewer instances of severe violence by the staff than control bars with similar characteristics. Although it is likely some people will act aggressively regardless of what precautions are taken, these findings indicate that modifying the social and physical atmosphere in a bar can help to reduce the likelihood of violence.

### Violence in the Home: Intimate Partner Violence

Research on intimate partner violence (IPV) demonstrates a high rate of co-occurrence of violence and alcohol use by one or both partners. In a representative sample of American families, Kaufman Kantor and Strauss (1990) found that heavy drinking by the husband was associated with husband-to-wife marital violence, independent of social class. Long-term studies (i.e., longitudinal research) also have shown a relationship between a husband’s drinking early in marriage and husband-to-wife violence later in the marriage. In a sample of newlyweds under 30 years of age, husbands who were heavier drinkers before marriage were more likely to be violent toward their wives in the first year of marriage (Leonard and Senchak 1996). A husband’s heavy premarital drinking also was predictive of severe violence in relationships that were high in conflict, but not in low-conflict relationships (Quigley and Leonard 1999). This, again, is consistent with the view of alcohol as a facilitator rather than an instigator of aggressive behavior.

Rates of IPV vary by race and ethnicity, as does the relationship of alcohol use to IPV. According to data from the 1995 National Study of Couples, the rate of IPV among African American couples is approximately 30 percent, more than twice as high as the rate among European American couples (11.5 percent) and also higher than the rate among Hispanic couples (17 percent) (Caetano et al. 2001). The relationship of alcohol use to IPV also varies. Nineteen percent of European American husbands and 24 percent of Hispanic husbands who drank at least five drinks a week committed IPV, as opposed to 40 percent of African American husbands who drank (Caetano et al. 2001). Differences between race/ethnic groups suggest that factors may predispose some people to both drinking and violence; however, these predispositions likely act in concert with the psychopharmacological effects of alcohol.

A number of studies have examined the role that alcohol plays in IPV among young adults. The Buffalo Newlywed Study compared couples who experienced only verbal aggression, only moderate aggression, or only severe aggression and found that drinking by husbands was more likely to occur in instances of severe physical violence than in instances of moderate physical violence or verbal aggression (Leonard and Quigley 1999). Among couples who had experienced both verbal and physical aggression, drinking by the husband was more likely in instances of physical violence than in instances of verbal aggression (Leonard and Quigley 1999). Testa and colleagues (2003) reported that more acts, and more severe acts, of violence occurred when the husbands had been drinking than when the husbands had not been drinking. In addition, some limited evidence suggests that wives are more likely to be physically aggressive when their husbands have been drinking. This aggressive behavior by the wives may be a reaction to aggression by the husbands, or the wives may behave aggressively on their own initiative (Testa et al. 2003).

Although not focused on a young adult population, research on treatment also suggests a relationship between acute alcohol use and the occurrence of IPV. This research suggests a causal relationship between drinking and the occurrence of a violent incident on the same day. Murphy and colleagues (2005) asked alcoholics and their spouses to report on conflict episodes and whether they involved physical violence. According to the wives’ accounts, alcoholic men were more likely to have been drinking during physically violent events. According to the husbands’ accounts, the men were likely to have consumed six or more drinks before these violent events. Similarly, Fals-Stewart (2003) assessed men entering treatment for alcoholism or domestic violence. He used a timeline followback method to determine the days on which alcohol abuse occurred and, independently, the days on which incidents of marital violence occurred. He found that the incidence of severe violence was much higher on days of heavy drinking (six or more drinks) than on days of no alcohol abuse, and that violence was most likely to occur within 4 hours of drinking. Several other studies have reported that alcohol use is more common at the time of serious physical assault events than near the time of less serious events (Martin and Bachman 1997; Thompson et al. 1999).

Finally, other evidence not specifically focused on young adults indicates that behavioral couples therapy for alcoholism reduces husband-to-wife marital violence. Prior to treatment, higher rates of IPV were found among samples of male alcoholics than in the general population. Significantly less violence occurred in the year after treatment compared with the year prior to marriage. In addition, the number of severely violent incidents decreased to rates similar to those seen in the general population (O’Farrell and Murphy 1995). More recent research has corroborated this early finding. O’Farrell and colleagues (2004), studying a sample of 303 male alcoholics undergoing behavioral couples therapy, found that reduction in husband-to-wife violence was correlated with greater treatment involvement. The more involved the males were in their alcoholism treatment, the less they drank and the less aggressive they were toward their spouses (O’Farrell et al. 2004).

## Conclusions

Young adults experience more violence than older age groups. Among young adult males, the most severe violence tends to occur in bars and clubs; young adult females are more likely to experience violence in the home. In both locations, the circumstances that provoke intoxicated aggression appear to arise from personality differences among people and from characteristics of the situations. People who are generally angry, impulsive, and less agreeable seem more likely to engage in intoxicated aggression. Bars with permissive atmospheres increase the probability of intoxicated aggression, and the more alcohol consumed, the greater the likelihood of injury. In domestic violence situations, alcohol use by the husband is predictive of severe violence only in marriages already high in conflict. All of these findings are consistent with the hypothesis that intoxication mainly serves to make conflict situations worse.

People often become intoxicated before getting into conflict. Evidence from experimental, survey, longitudinal, and event-based research suggests that alcohol intoxication contributes to violence. A better understanding is needed of the pharmacological effects of alcohol on the decisionmaking involved in aggressive interactions. Models such as alcohol myopia, which proposes that alcohol reduces attention to cues that inhibit aggression, and the anxiolysis disinhibition model, which proposes that alcohol dampens the anxiety associated with inhibitory cues, provide useful frameworks for a better understanding of intoxicated aggression. However, research has not yet identified which model provides the best explanation. Although much has been discovered about the relationship between alcohol use and violence, much research remains to be done. More understanding of alcohol’s effects on people with different propensities toward aggressive behavior is needed. Individual differences in hostility, anger, impulsiveness, agreeableness, and alcohol expectancies have been identified as important, but it still is not clear how and why people with these characteristics seem to be more likely to engage in intoxicated aggression. A fuller understanding of these processes will help inform more effective approaches to preventing and treating alcohol-involved violent behavior.
